# Orbital manifestations of Langerhans Cell Histiocytosis: A report of three cases

**DOI:** 10.4103/0974-620X.57315

**Published:** 2009

**Authors:** Jayanta K. Das, Ronel Soibam, B. K. Tiwary, D. Magdalene, S. B. Paul, Cida Bhuyan

**Affiliations:** Department of Ophthalmology, Sri Sankaradeva Nethralaya, Guwahati; 1 Department of Life Science and Chemistry, Assam University Silchar; 2 Department of Oncology, B.Borooah Cancer Institute, Guwahati, India

**Keywords:** *Computerized tomography*, *histopathological examination*, *Langerhans cell histiocytosis*, *ultrasonography*

## Abstract

Langerhans Cell Histiocytosis (LCH) is a spectrum of disorders characterized by accumulation of histiocytes in various tissues. It is rarely encountered in ophthalmic practice and has an affinity for the orbit. We report three patients with LCH involving the lateral orbital wall, each with a different form of the condition.

## Introduction

Langerhans Cell Histiocytosis (LCH) is an uncommon multisystem disorder of unknown etiology, characterized by accumulation of histiocytes in various tissues. It has a variable clinical course, and although is occasionally seen in adults, predominantly occurs in children.[1]

Three clinical forms of LCH are recognized, namely, Eosinophilic granuloma, Hand–Schuller–Christian disease and Letterer–Siwe disease. Eosinophilic granuloma is characterized by the presence of histiocytic unifocal or multifocal mass lesions that generally originate in the bone. Hand–Schuller–Christian disease manifests as a triad of exophthalmos, bony defects of skull and diabetes insipidus.[2] Letterer–Siwe disease is characterized by widespread soft tissue and visceral involvement with or without bone lesions. It has an acute or subacute course, and is sometimes fatal.

LCH is classified into 4 groups based on a clinical staging system i.e. Group A – bone only or bone and contiguous soft tissue involvement, Group B – skin or other squamous mucus membranes only or with involvement of related superficial lymph nodes, Group C – soft tissue and viscera only and Group D – multisystem disease.[1,3] In order to standardize the classification of this disorder, a pathologic staging has also been proposed.[3] We report three children with orbital LCH.

## Case Reports

### Patient 1

**Figure 1:**
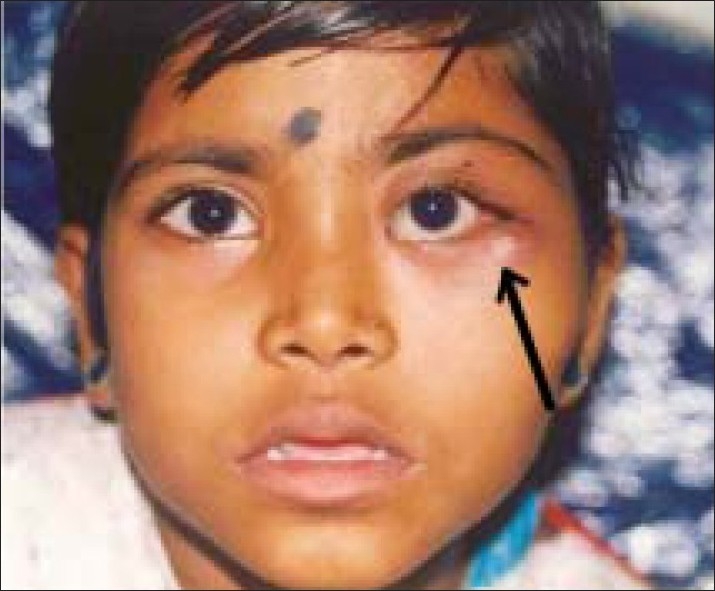
Patient 1 – LCH presented with ill-defined mass in lateral orbital wall

**Figure 2:**
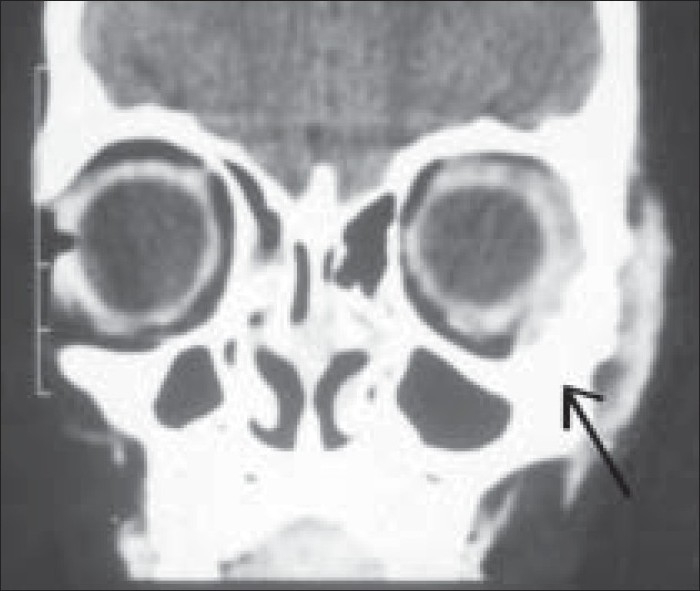
Patient 1 – CT scan of the orbit showing an infiltrative mass with irregular margins arising from the lateral orbital wall

**Figure 3:**
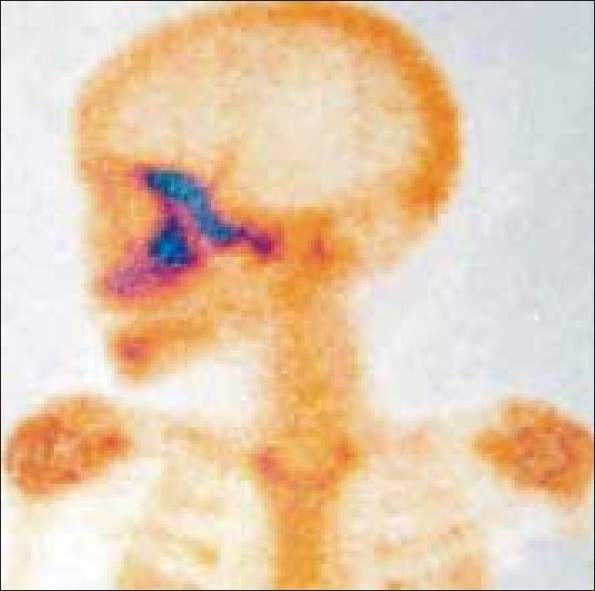
Patient 1 – Radio isotope bone scan revealing an increased uptake in the lateral orbital wall

An eight-year-old girl presented with a painless, slowly progressive swelling in the lateral aspect of the left orbit of three months duration. On examination, visual acuity was 20/20 in both eyes (OU). There was a 3 mm proptosis of the left eye (OS) with restriction of abduction [Figure 1]. Palpation revealed a hard non-tender mass over the zygomatic bone. Systemic examination was normal except for raised absolute eosinophil count of 450 cell/mm. Computerized Tomography (CT) scan of orbit showed an infiltrative mass with irregular margins arising from left lateral orbital wall [Figure 2]. Radioisotope bone scan revealed an increased uptake of the involved bone [Figure 3]. Based on the clinical picture an infiltrative or neoplastic lesion was suspected. Curettage of the lateral orbital wall was done. Histopathological examination revealed the characteristic Langerhans cell – a large ovoid mononuclear cell, 15–25 μm in diameter, with a discrete nucleolus, slightly eosinophilic homogenous cytoplasm and Birbeck granules. The diagnosis of LCH was further confirmed by positive S100 protein and adenosine triphosphatase antibody staining on immunohistochemical studies.

**Figure 4:**
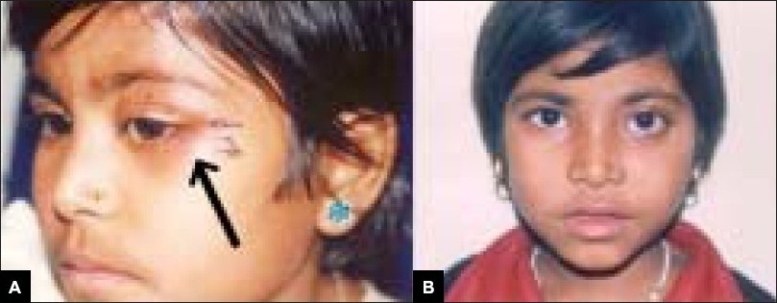
Patient 1 – A) Early postoperative view following curettage B) Late postoperative view showing resolution

The patient received external radiotherapy total of 1500 cGy in three fractions, as it was a painful lesion along with significant soft tissue involvement. Intralesional steroid had not been used intraoperatively. She has been followed up for a period of four years without any recurrence [Figure 4].

### Patient 2

**Figure 5:**
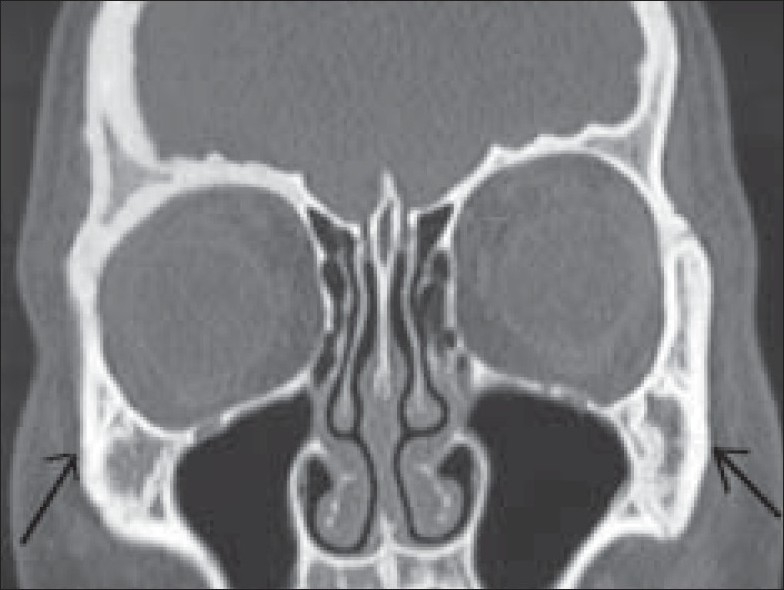
Patient 2 – CT scan of the orbit showing osteolytic lesion in both lateral orbital wall

A seven-year-old female reported with periocular fullness OU. There was a bilateral proptosis of 3 mm right eye (OD) and 2 mm OS, respectively, with a palpable rubbery mass over the lateral wall of the orbit OU. The periocular cutaneous tissue and cervical lymph node was also involved on the left side. Ocular motility and ocular evaluation was unremarkable. Systemic examination revealed splenomegaly, which was confirmed by abdominal ultrasonography (USG). Routine blood examination did not show any abnormality except raised erythrocyte sedimentation rate (ESR) and eosinophil count. CT scan of the orbit showed bilateral lytic lesions of lateral orbital wall [Figure 5]. Curettage biopsy of the orbital mass was done in the right side. Histopathological examination and S100 protein test were consistent with a diagnosis of LCH. The presence of splenomegaly favored a diagnosis of Letter–Siwe disease. Considering the multisystem involvement, she was treated with radiotherapy (1000 cGy) in two fractions for orbital bony lesion and chemotherapy (vinblastine 6 mg/m^2^) to take care of the extra orbital involvement. Reduction in the size of spleen and lytic lesions of the bone were noted following therapy.

### Patient 3

A three-year-old male child presented to us with swelling around the right periorbital area. There was a 5 mm eccentric proptosis, severe ptosis and cutaneous involvement of the surrounding periocular tissue OD. Pre-auricular lymphadenopathy with purulent discharge from the right ear was noted. Systemic examination revealed both splenomegaly and hepatomegaly, which was confirmed by abdominal ultrasound. CT scan of the orbit revealed degenerative cystic lesions in the right orbit over the temporal and maxillary region with extensive soft tissue involvement over the fontal, mastoid, maxillary and mandibular regions.

**Figure 6:**
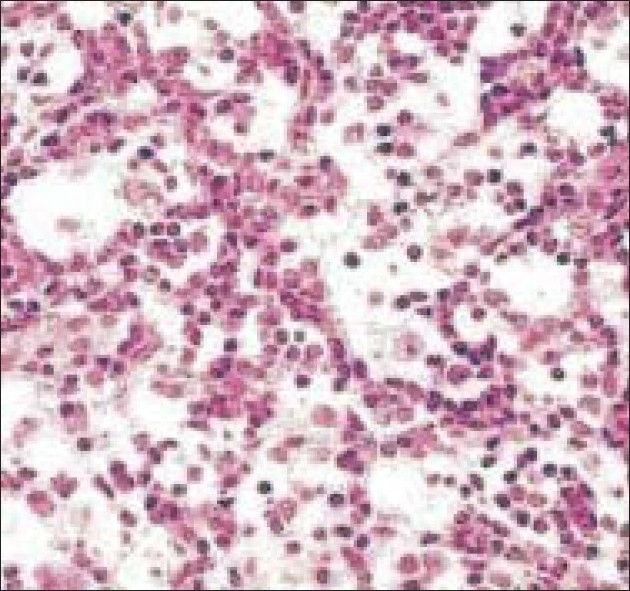
Patient 3 – Histopathologic picture on high magnification showing numerous histiocytes and eosinophils

Histopathological examination of biopsy of the cutaneous lesion [Figure 6] confirmed the diagnosis of LCH. This diagnosis was further consolidated by the S-100 protein and adenosine triphosphatase antibody tests. The patient was treated with by 12 cycles of chemotherapy (vinblastine 6 mg/m^2^) along with systemic corticosteroid (prednisolone). Following chemotherapy the periocular swelling subsided with reduction of cystic bony changes. The patient has been followed up for last five years without any recurrence.

## Discussion

LCH is an uncommon cause of orbital tumor. The incidence of orbital involvement in LCH has been reported to vary from 1 to 20%, and usually presents as proptosis, as was seen in our patients.[1,4,5]

The international Histiocytic society defined objective criteria for diagnosing LCH.[6] According to the society, two or more of the following are required for a diagnosis: positive staining for adenosine triphosphate, S-100 protein antibody, alphamannosidase or peanut lectin binding.

In children with both orbit and systemic involvement, it is easier to take biopsy from cutaneous tissue, as was done in one of our patients (Patient 3), while therapeutic curettage was performed in Patients 1 and 2.

The treatment for LCH is controversial because of the unpredictable outcome following therapy and occurrence of spontaneous healing in some cases.[7] Various treatment options include close observation, surgical curettage, local injection of corticosteroids, low dose radiotherapy, high dose systemic corticosteroids, chemotherapy, and for more recalcitrant cases, bone marrow transplantation and antibody therapy.[8] The choice of therapeutic regime is based ultimately on disease severity, and number of systems involved.

**Single System Disease:** Solitary bone lesions are treated locally with curettage or excision. Painful bone lesions may require intralesional steroid or rarely radiation therapy. In cases with severe cutaneous involvement, topical nitrogen mustard may be used. Treatment resistant sites may require systemic chemotherapy.

**Multisystem Disease:** Systemic chemotherapy is indicated for multisystem disease. Low to moderate doses of methotrexate, prednisone and vinblastine are used. Thalidomide has been proposed as an agent for treating refractory/relapsing disease. In patients with single bony lesion, biopsy and curettage may be followed by complete resolution.[9] Chemotherapy–corticosteroid regimen used in multisystem involvement is not effective for bone involvement, but reduces the size of the mass. No definite criteria have been established regarding radiation in orbital histiocytosis; however, a short course of low dose radiation under the supervision of pediatric onco-radiologist may be given to induce remission.[8]

The prognosis and ultimate outcome depends not only on the therapy; but upon several other factors such as age of presentation, multisystem involvement and response to initial therapy. The mortality rate in children and patients with multisystem involvement is high because of failure of key organs such as bone marrow, lungs and liver. The five-year survival rate in children under two years, even with aggressive chemotherapy is only 50%.[10] However, Eosinophilic granuloma, the most common form of LCH, carries a favorable prognosis and requires minimal intervention.[11]
